# The FASD Eye Code: a complementary diagnostic tool in fetal alcohol spectrum disorders

**DOI:** 10.1136/bmjophth-2021-000852

**Published:** 2021-10-22

**Authors:** Eva Aring, Emelie Gyllencreutz, Valdemar Landgren, Leif Svensson, Magnus Landgren, Marita Andersson Grönlund

**Affiliations:** 1 Department of Clinical Neuroscience, Institute of Neuroscience and Physiology, Sahlgrenska Academy at the University of Gothenburg, Gothenburg, Sweden; 2 Deparment of Ophthalmology, Region Västra Götaland, Sahlgrenska University Hospital, Mölndal, Sweden; 3 Department of Paediatrics, Unit of Neurodevelopmental Disorders, Region Västra Götaland, Skaraborg Hospital, Mariestad, Sweden; 4 Gillberg Neuropsychiatry Centre, Institute of Neuroscience and Physiology, University of Gothenburg, Gothenburg, Sweden; 5 Department of Ophthalmology, Faculty of Medicine and Health, Örebro University, Örebro, Sweden

**Keywords:** child health (paediatrics), optics and refraction, vision, retina, diagnostic tests/investigation, optic nerve, public health

## Abstract

**Objective:**

To create an easy-to-use complementary ophthalmological tool to support a fetal alcohol spectrum disorder (FASD) diagnosis.

**Methods and Analysis:**

The FASD Eye Code was derived from 37 children with FASD evaluated along with 65 healthy age-matched and sex-matched controls. Four ophthalmological categories, which are abnormalities commonly found in children with FASD, were ranked independently on a 4-point scale, with 1 reflecting normal finding and 4 a strong presence of an abnormality: visual acuity, refraction, strabismus/binocular function and ocular structural abnormalities. The tool was validated on 33 children with attention deficit/hyperactivity disorder (ADHD), 57 children born moderate-to-late premature (MLP) and 16 children with Silver-Russell syndrome (SRS). Among children with ADHD none was born prematurely or small for gestational age (SGA) or diagnosed with FASD. Among children born MLP none was SGA, had a diagnosis of ADHD or FASD, or a history of retinopathy of prematurity. Children with SRS were all born SGA, half were born preterm and none had FASD. Children with FASD were re-examined as young adults.

**Results:**

An FASD Eye Code cut-off total score of ≥10 showed an area under the curve (AUC) of 0.78 (95% CI 0.69 to 0.87), with 94% specificity and 43% sensitivity, in discriminating between FASD and controls, MLP and ADHD, corresponding to a positive likelihood ratio (LR+) of 7.5. Between FASD and controls, an AUC of 0.87 (CI 0.80 to 0.95), with 100% specificity and 43% sensitivity, was found; between FASD and SRS, an AUC of 0.60 (CI 0.45 to 0.75) was found, with 88% specificity and 43% sensitivity. A cut-off score of≥9 showed a specificity of 98% and a sensitivity of 57% for FASD versus controls, corresponding to an LR+ of 36.9. Scores in individuals with FASD were stable into young adulthood.

**Conclusion:**

The FASD Eye Code has the potential to serve as a complementary tool and help to strengthen an FASD diagnosis.

**Key messages**

**What is already known about this subject?**

- Fetal alcohol spectrum disorders (FASD) are a global health concern and many different diagnostic criteria are used worldwide.
- Many children prenatally exposed to alcohol go undiagnosed and related ophthalmological impairments that are amenable to interventions are overlooked.

**What are the new findings?**

- The FASD Eye Code is created as a complementary tool to strengthen a suspected FASD diagnosis, irrespective of which specific FASD detecting criteria are used.

**How might these results change the focus of research or clinical practice?**

- The FASD Eye Code, which is easy to use both clinically and in fieldwork, may support the criteria for diagnosing individuals with suspected FASD and help identify children with treatable ophthalmological problems.
- These are problems that—if identified in a timely manner—can be addressed to prevent additional lifelong impairments in these already affected individuals with FASD.

## Introduction

**Figure 1:**
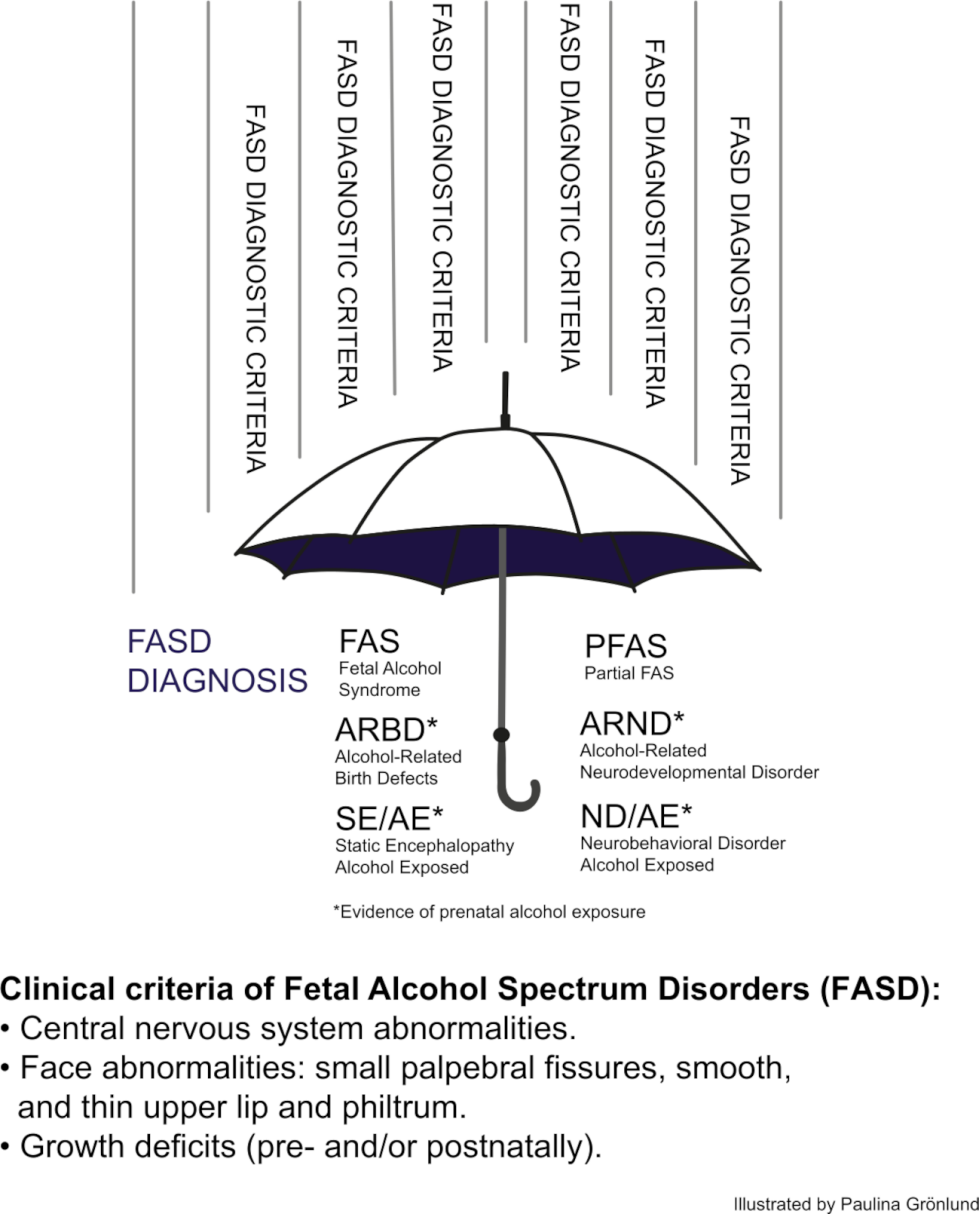
Different subgroups under the umbrella term fetal alcohol spectrum disorders. Figure published with permission from Paulina Grönlund.

Since the recognition of fetal alcohol syndrome (FAS), alcohol has been shown to produce a wide spectrum of physical, neurological, cognitive and ophthalmological aberrations, now known as fetal alcohol spectrum disorders (FASD).^1–15^ The prototypical features of FAS are small palpebral fissures, a thin upper lip, a smooth philtrum, growth restrictions and central nervous system abnormalities (figure 1).^1–3 8 16–18^ Although previous studies have shown that small palpebral fissures are not the only ophthalmological features occurring frequently in children with FASD,^9 11 13 19^ the role of ophthalmological assessment in the work-up of FASD may be underestimated.

Alcohol use during pregnancy is a public health problem worldwide. A meta-analysis estimated that over 100 000 children around the world are born with FAS every year.^20^ The incorporation of the diagnosis ‘neurodevelopmental disorder – prenatally exposed’ into the *Diagnostic and Statistical Manual of Mental Disorders, Fifth Edition* is a recognition of the association between alcohol exposure and behavioural and cognitive deficits. Within this wider spectrum of prenatal alcohol exposure (PAE), there is less agreement on what constitutes an alcohol-related aetiological diagnosis,^21 22^ and several sets of diagnostic criteria are used around the world to diagnose FASD. However, the key features of FAS in each set of criteria are the same: growth deficiency, facial dysmorphology and neurobehavioural impairment (figure 1).^2 3 16–18^

Ophthalmological findings, such as subnormal visual acuity, refractive errors, motility disorders, strabismus, and abnormalities of the retinal vessels and optic head, are frequently found in individuals with FASD, but these ophthalmological findings are also found in children with, for example, attention deficit/hyperactivity disorder (ADHD), as well as prematurely born children.^23 24^ On the other hand, both ADHD and prematurity are frequently found in children diagnosed with FASD.^4 5 8^ To what extent this co-occurrence is caused by confounding alcohol exposure is unknown, and clinically discriminating between alcohol exposure and other factors remains a challenge. Strabismus and refractive errors may cause amblyopia and visual impairment, which are treatable if found in early childhood, but if not treated may present additional problems for already affected individuals. Moreover, other groups with neurodevelopmental syndromes and genetic disorders may also present with similar ophthalmological abnormalities, although other clinical symptoms are usually present as well.

The medical diagnostic process is probabilistic in nature.^25^ Arriving at a seemingly dichotomous ‘yes or no’ answer to a diagnosis is guided by a probabilistic weighing of history, findings and tests, where the overarching question is: ‘What is the probability that the patient has this diagnosis, given the information and results?’ In this process, information and tests unspecific to the diagnosis in question help to adjust the probabilities of the diagnosis in a useful way. Analogous to supportive laboratory investigations, a complementary eye diagnostic tool could provide independent verification for clinicians, thus strengthening and helping FASD diagnostics. However, to be of use, findings must differentiate between FASD and children with other conditions presenting with similar symptoms. It should also be considered that anthropometric criteria are less evident in adulthood, while cognitive impairment and psychiatric morbidity are more evident in adults with FASD.^4 5 26 27^

The aims of this study were threefold: first, to develop and propose a complementary and easy-to-use tool based on ophthalmological findings to support an FASD diagnosis; second, to validate the FASD Eye Code’s capacity to discriminate FASD eye findings from prematurity, growth restriction and ADHD without diagnosed FASD; and third, to test the tool in long-term follow-up of individuals with FASD from childhood to young adulthood. In addition, the tool should be useful both in eye clinics and elsewhere, allowing use in outreach and epidemiological surveys of FASD in different communities, while simultaneously identifying treatable deficits requiring management no matter what the cause.

## Materials and methods

### Developing the FASD Eye Code

**Figure 2:**
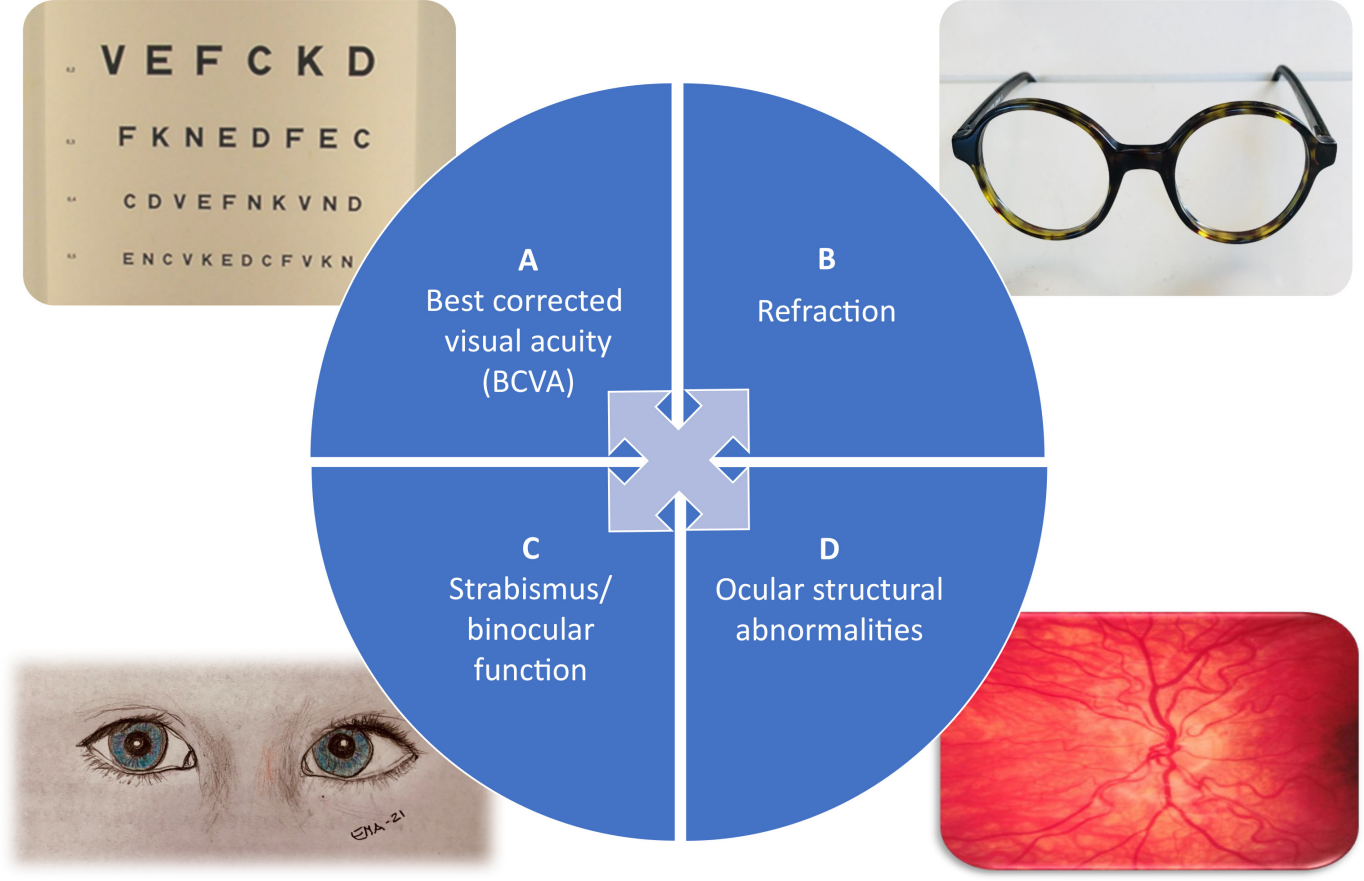
The FASD Eye Code categories: (A) best corrected visual acuity, (B) refraction, (C) strabismus and binocular function (drawing by Eva Aring), and (D) ocular structural abnormalities. FASD, fetal alcohol spectrum disorder.

Based on the accrued evidence of ophthalmological studies on FASD and our own clinical experience,^4 5 9–14 19^ four of the most commonly affected ophthalmological features in children with FASD were chosen to create the four categories constituting the FASD Eye Code. The four categories are (A) best corrected visual acuity (BCVA), (B) refraction, (C) strabismus and binocular function, and (D) ocular structural abnormalities. By design, the selected tests are easy to use both in the clinic and on the field (figure 2).

Among the chosen ophthalmological features, two are structural measures (refraction and ocular structural abnormalities) and two are functional tests (BCVA and strabismus/binocular function). The functional measurements represent both afferent and efferent functions. Normal scores in each category are based on known levels in a normal paediatric population,^28 29^ and the maximal scores are based on affected ophthalmological features well characterised in children with FASD.^9–14 30 31^ We have excluded palpebral fissure length in the FASD Eye Code since this variable is included in all FASD diagnostic criteria previously mentioned.^2 3 16–18^

### Definitions and scores of the FASD Eye Code

- BCVA: visual acuity was tested at 3 m with the best possible correction and with a linear Konstantin Moutakis (KM)-Boks chart.^31^
- Refraction: refraction was tested under cycloplegia and was performed with an autorefractor (Topcon A6300/KR8800; Topcon Corporation, Tokyo, Japan) after a single instillation of a mixture of cyclopentolate (0.85%) and phenylephrine (1.5%).
- Strabismus and binocular function: strabismus was diagnosed using a cover test and was defined as a deviation that is manifested always (heterotropia) or intermittently, or as a latent deviation (heterophoria).^29^ Binocular function was performed primarily with the Nederlandse Organisatie voor Togepast Natuurwetenschappelijk Onderzoek (TNO) random dot stereo test. Lang I was used if the child could not participate in the TNO test.
- Ocular structural abnormalities: ptosis, epicanthal folds and ocular fundus were examined clinically, then later by indirect ophthalmoscopy. Fundus photos were taken.

On a 4-point scale, each feature is ranked independently, with ‘1’ reflecting normal ophthalmological findings and ‘4’ reflecting the presence of an abnormality commonly found in individuals with FASD. Thus, ‘4444’ represents the most severe expression of reduced BCVA, significant refractive errors, manifest strabismus or defect binocular functions, and structural abnormalities of the eye. At the opposite end of the scale, code ‘1111’ represents normal ophthalmological findings. The four categories included in the FASD Eye Code and the definitions of the scores (1–4) are summarised in the protocol used for evaluating the FASD Eye Code (online supplemental file 1).

10.1136/bmjophth-2021-000852.supp1 Supplementary data

### Study design and participants

Altogether, the FASD Eye Code was evaluated on 208 different individuals with FASD (n=37), ADHD (n=33), born moderate-to-late preterm (MLP) (n=57) and Silver-Russell syndrome (SRS) (n=16) and in controls (n=65). Children with FASD were drawn from a population-based study of 71 children adopted from Eastern Europe and examined at a mean age of 7.5 years (online supplemental file 2).^4 32^

10.1136/bmjophth-2021-000852.supp2 Supplementary data

- Group 1 (n=37): 15 female, 22 male, mean age 9.8 years (range 4.9–10.5 years), diagnosed with FASD according to the Institute of Medicine criteria,^1^ including the following subgroups: FAS (n=21), partial FAS (PFAS) (n=10) and alcohol-related neurodevelopmental disorder (ARND) (n=6).^4^
- Group 2 (n=65): 27 female, 38 male, mean age 9.9 years (range 4.1–12.3), healthy children who served as a control group and were matched by age and sex. Controls for the FASD group were selected from a group of 143 healthy Caucasian school children living in the same area. No child was born small for gestational age (SGA) or prematurely or had a diagnosis of ADHD or FASD.^28^

The FASD Eye Code was then validated on the following additional groups (online supplemental file 2).

- Group 3 (n=33): 12 female, 21 male, mean age 12.1 years (range 6.3–17.5), diagnosed with ADHD. No child was born SGA or prematurely or diagnosed with FASD.^24^
- Group 4 (n=57): 23 female, 34 male, mean age 5.7 years (range 5.4–5.9), born MLP between 32 and 36 weeks’ gestation. No child was born SGA, had a diagnosis of ADHD or FASD, or had a history of retinopathy of prematurity (ROP). These children were selected from a previous study of 78 children, as described in detail elsewhere.^24^
- Group 5 (n=16): 8 female, 8 male, mean age 11.2 years (range 3.4–18.1), with SRS. Half of the children were born preterm and all were born SGA. None was diagnosed with FASD.^33^

Patients or the public were not involved in the design, or conduct, or reporting or dissemination plans of our research.

### The FASD Eye Code: long-term follow-up

**Figure 3:**
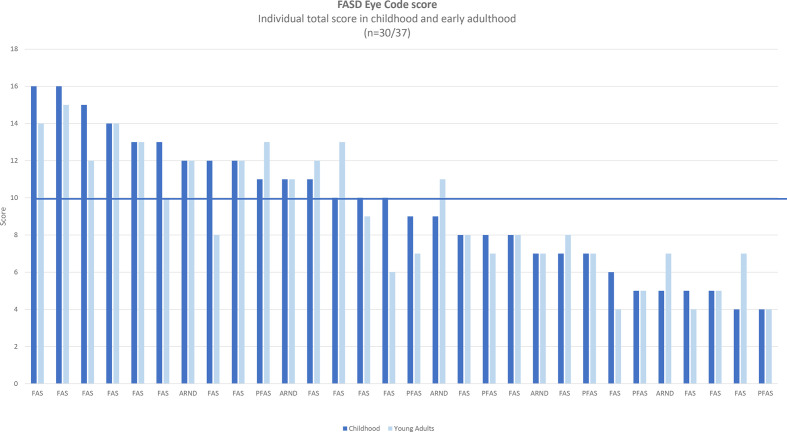
Individual FASD Eye Code total score in 30 subjects diagnosed with FASD, in both childhood and early adulthood. ARND, alcohol-related neurodevelopmental disorder; FAS, fetal alcohol syndrome; FASD, fetal alcohol spectrum disorders; PFAS, partial FAS.

In total, 30 out of the 37 young adults (81%) with FASD (FAS=19, PFAS=6, ARND=5) participated in a follow-up investigation at a mean age of 23 years (range 19–28 years). Six young adults declined to participate and one discontinued the study (figure 3).^12^

### Statistical analysis

SPSS V.22 and/or SAS V.9.2 were used for analyses of mean, median, range, percentages, CI and IQR and were calculated for descriptive purposes. When analysing two related samples, the Wilcoxon signed-rank test was used. To test the specificity and sensitivity of the FASD Eye Code in differentiating FASD from other conditions, we performed receiver operating characteristic (ROC) curve analyses in R V.3.6.3 (R Core Team 2020), mainly the pROC package, an open-source package for R and S+ used to analyse and compare ROC curves (http://www.biomedcentral.com/1471-2105/12/77). To examine the diagnostic utility, we extracted diagnostic indices (sensitivity, specificity, accuracy, and negative and positive likelihood ratio) by cut-off values for the FASD Eye Code.

A control group of healthy Swedish school-aged children living in the same area were selected using the population matching method by minimising the maximal t-values.^34^ This method matches two populations by iteratively selecting individuals from a reference population with the minimum t-test score,^28^ tested against the FASD group with respect to age and sex.

## Results

Table 1 shows the median (IQR) FASD Eye Code score of the four categories (BCVA, refraction, strabismus and binocular function, and ocular structural abnormalities) in all groups (FASD, age-matched and sex-matched controls, ADHD, MLP and SRS) and FASD subgroups (FAS, PFAS and ARND), as well as the total median scores (range, IQR) for all groups.

**Table 1:** The FASD Eye Code median score in the four categories and the total score among the five groups

Groups		FASD Eye Code score in different categories Median (IQR)				FASD Eye Code total score Median (range), IQR
		A BCVA	B Refraction	C Binocular function/ strabismus	D Ocular structural abnormalities	
Group 1: FASD (n=37)		1.0 (2.0)	2.0 (3.0)	3.0 (3.0)	3.0 (2.0)	9 (16–4), 4.5
	FAS (n=21)	2.0 (1.5)	1.0 (3.0)	3.0 (1.5)	3.0 (1.5)	10 (16–4), 5.5
	PFAS (n=10)	1.5 (1.0)	1.5 (1.5)	1.0 (2.25)	2.5 (2.0)	7.0 (11–4), 3.25
	ARND (n=6)	2.0 (1.25)	1.0 (0.25)	2.5 (3.0)	2.0 (2.0)	7.5 (12–4), 6.5
Group 2: controls (n=65)		1.0 (0.0)	1.0 (0.0)	1.0 (0.0)	1.0 (0.0)	4.0 (9–4), 0.0
Group 3: ADHD (n=33)		1.0 (0.0)	1.0 (2.0)	1.0 (2.0)	1.0 (2.0)	7.0 (11–4), 4.5
Group 4: MLP (n=57)		1.0 (1.0)	1.0 (1.0)	1.0 (1.0)	1.0 (0.0)	5.0 (13–4), 2.5
Group 5: SRS (n=16)		1.0 (1.0)	1.5 (3.0)	1.0 (1.75)	3.0 (2.0)	7.0 (13–4), 3.0

ADHD, attention deficit/hyperactivity disorder; ARND, alcohol-related neurodevelopmental disorder; BCVA, best corrected visual acuity; FAS, fetal alcohol syndrome; FASD, fetal alcohol spectrum disorders; MLP, moderate-to-late preterm; PFAS, partial FAS; SRS, Silver-Russell syndrome.

Figure 3 shows the individual total score, in both childhood and early adulthood, for the 30 subjects diagnosed with FASD.

**Figure 4:**
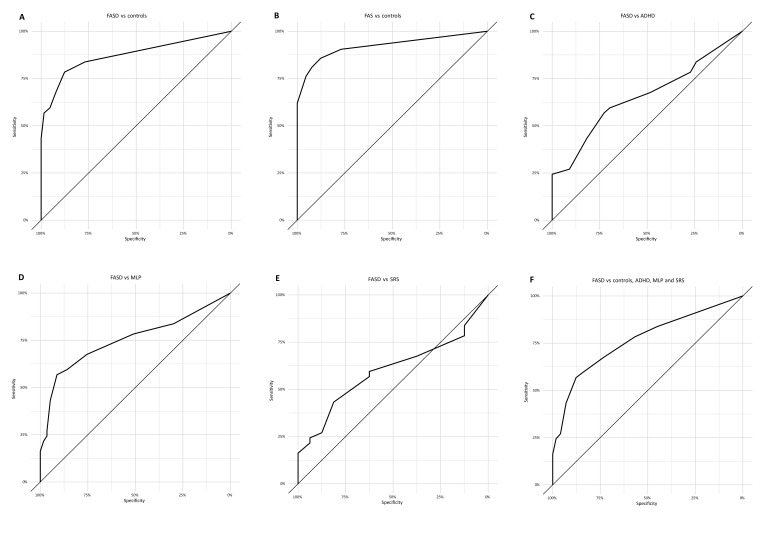
Six receiver operating characteristic curve analyses between the different groups, comparing children with (A) FASD and their age-matched and sex-matched controls; (B) FAS versus controls; (C) FASD versus ADHD; (D) FASD versus MLP; (E) FASD versus SRS; and (F) FASD versus controls, ADHD, MLP and SRS. ADHD, attention deficit/hyperactivity disorder; FAS, fetal alcohol syndrome; FASD, fetal alcohol spectrum disorder; MLP, moderate-to-late premature; SRS, Silver-Russell syndrome.

A cut-off total score of ≥10 was chosen to represent an FASD diagnosis based on the total sample (this score was obtained by 16 of 37 participants with FASD, 6 of 33 with ADHD, 3 of 57 with MLP, 3 of 16 with SRS and 0 of 65 controls). Thus, an FASD Eye Code cut-off score of ≥10 has 100% specificity and 43% sensitivity, with an area under the curve (AUC) of 0.87 (95% CI 0.80 to 0.95), in discriminating between FASD and healthy controls. When comparing FASD versus controls, ADHD and MLP, the specificity was 94% and the sensitivity was 43% (AUC=0.78; 95% CI 0.69 to 0.87). This result corresponds to a positive likelihood ratio of 7.5. If comparing FASD versus all groups, the AUC was 0.76 (95% CI 0.67 to 0.86). When comparing FASD versus ADHD, the AUC was 0.66 (95% CI 0.53 to 0.78); for FASD versus MLP, the AUC was 0.75 (95% CI 0.64 to 0.86); and for FASD versus SRS, the AUC was 0.60 (95% CI 0.45 to 0.75). The subgroup FAS versus controls showed the highest AUC value of 0.92 (95% CI 0.85 to 1.0), and for a cut-off score of ≥10 the specificity was 100% and the sensitivity was 62%. A cut-off score of ≥9 showed a specificity of 98% and a sensitivity of 57% for FASD versus healthy controls, corresponding to a positive likelihood ratio of 36.9. Tables of diagnostic indices for ROC curves (cut-off, sensitivity, specificity, accuracy, likelihood ratio) are provided in (figure 4A–G and online supplemental file 3).

10.1136/bmjophth-2021-000852.supp3 Supplementary data

## Discussion

We created and evaluated a new complementary diagnostic tool, the FASD Eye Code, by comparing children with FASD, age-matched and sex-matched healthy controls, and groups of individuals with ADHD, MLP and SRS. Long-term follow-up (re-examination) of individuals with FASD in young adulthood indicated the persistence of childhood findings. Our results show that the FASD Eye Code can distinguish among different FASD subgroups and discriminate between different patient groups with similar ophthalmological problems.^23 24 33^ The FASD Eye Code shows better specificity than sensitivity for FASD, with a total score of ≥10 having an increasing likelihood of ruling in an FASD diagnosis. In addition, our results are of clinical importance and require management no matter what the cause is, and the findings may offer useful clues to potential aetiology. To the best of our knowledge, this is the first ophthalmological tool developed to support an FASD diagnosis in both childhood and young adulthood.

The wide range of scores among children with FASD is not surprising as it reflects the range of eye findings associated with PAE during the different stages of development of the fetus, and in some cases eye findings may be due to PAE without rising to a FASD diagnosis. Thus, children who scored ≥10 were most commonly those with fully developed FAS, and no one among the controls scored 10 or above. Among children with SRS, 2 of 16 children scored 11 and 1 scored 13. Unlike the other evaluated groups (ADHD, MLP and controls), all children with SRS showed growth deficits both prenatally and at the time of assessment, which are among the hallmarks of an FASD diagnosis. However, children with SRS have other symptoms and signs, including ophthalmological findings,^33^ which indicate an SRS rather than an FASD diagnosis. The same is mostly true when differentiating FASD from other syndromes.^12^ However, genetic testing could also be valuable in these cases as a co-occurrence with PAE is possible.

Six children in the ADHD group (children without diagnosed FASD nor born preterm/SGA) were noted to have an FASD Eye Code total score ≥10; three of these children with ADHD had a score of 11 or above. This may be misclassification due to confounding from undiagnosed PAE and undiagnosed FASD, or due to neurodevelopmental afflictions of other aetiology. The criteria for ADHD, a symptom-based diagnosis, were met by the majority of children/young adults with an aetiological diagnosis of FASD.^5 8^

Visual acuity and refraction are age-dependent,^28^ which must be considered when diagnosing different individuals and age groups. No gender differences between the ophthalmological variables used in the code have been shown in controls.^28^ However, refraction and strabismus may have ethnicity-based variation.^35^ Thus, our values are suitable for Caucasian children between 4 and 15 years of age. Since the typical facial feature of FAS seems to diminish with age,^27^ we tested whether the FASD Eye Code could still be applicable in young adults with FASD. Out of 37 individuals, 30 were assessed in both childhood and young adulthood,^12^ showing no significant differences in the FASD Eye Code total score. Since refraction differs in different age groups, myopia was also evaluated in the young adult group, with a cut-off of ≥2.0 dioptre (D) spherical equivalent (SE). However, no difference was noted in the FASD Eye Code total median score when comparing the two different cut-offs (myopia ≥1 D SE vs ≥2 D SE), as individuals with FASD more often have astigmatism or anisometropia, which gives a higher score in the refraction category.

### Strengths and limitations

The strength of the study is that the same ophthalmological methods were used in all groups. All the ophthalmological tests necessary for using the FASD Eye Code are well known, inexpensive and can easily be used in low-resource settings outside the clinic. Furthermore, as technology develops, handheld instruments may provide more accurate investigations in the future. To minimise confounding, no children with ROP were included in the study.

Children with ADHD, children born MLP and children with SRS may present with ophthalmological findings similar to those of children with FASD. Since FASD is an aetiological diagnosis, it warrants consideration in children presenting with prematurity, SGA and ADHD. Even so, the FASD Eye Code gave these groups lower scores—a result that provides support to the use of the code. The same experienced multidisciplinary team investigated all the children in the different groups, with a meticulous and consistent methodology.^3 4 11 12 24 25 29 30 33 34^

Our study has some limitations: the small size of the groups, the fact that the ophthalmological findings in isolation are unspecific and that the eye code was derived from a group of adopted children from Eastern Europe with FASD. In addition, other confounding factors may have had an impact on the results which must be addressed when planning future studies.

In 35 years working with children exposed to alcohol, we have learnt that PAE is under-recognised and the dosimetry complicated. Thus, our control group may include individuals exposed to alcohol without our knowledge. Irrespective of the FASD diagnosis, this tool may identify treatable eye problems. Refractive errors are treatable with glasses, and if not treated in childhood may affect visual acuity for life; an untreated strabismus can also result in amblyopia and eye strain. The code needs to be independently validated by other assessors examining other FASD cohorts and comparison groups. Further studies are needed to validate the FASD Eye Code longitudinally—that is, within the same individuals with FASD and in other age groups with different ethnicities, as well as in syndromes of other aetiology and in healthy controls with a wider age range. We recommend using the code as a complement when there is suspicion for an FASD diagnosis and when genetic syndrome phenocopies have been ruled out.

In conclusion, in this derivation cohort, an FASD Eye Code total score ≥10 significantly corroborates an FASD diagnosis, although low scores cannot rule out FASD. The FASD Eye Code may support the criteria for diagnosing individuals with suspected FASD and help identify children and young adults with treatable ophthalmological problems. In addition, all the ophthalmological tests involved in the FASD Eye Code are well known, relatively inexpensive and can easily be used outside the clinic. In the future, as technology develops, handheld instruments such as different refractive and imaging devices may provide more accurate investigations. Ophthalmological assessment should be a routine part of the FASD work-up and the FASD Eye Code can help to highlight a need for intervention.

## Data Availability

Data are available upon reasonable request. All data relevant to the study are included in the article or uploaded as supplementary information.

## References

[1] Hoyme HE, May PA, Kalberg WO, et al. A practical clinical approach to diagnosis of fetal alcohol spectrum disorders: clarification of the 1996 Institute of medicine criteria. Pediatrics 2005;115:39–47. 10.1542/peds.2004-0259 15629980 PMC1380311

[2] Hoyme HE, Kalberg WO, Elliott AJ, et al. Updated clinical guidelines for diagnosing fetal alcohol spectrum disorders. Pediatrics 2016;138:e20154256–18. 10.1542/peds.2015-4256 27464676 PMC4960726

[3] Astley S. Prenatal alcohol use and fetal alcohol spectrum disorders: diagnosis, assessment and new directions in research and multimodal treatment. diagnosing fetal alcohol spectrum disorders (FASD). Illinois: Bentham Science Publishers, Ltd. Bentham eBooks, 2011: 3–29.

[4] Landgren M, Svensson L, Strömland K, et al. Prenatal alcohol exposure and neurodevelopmental disorders in children adopted from eastern Europe. Pediatrics 2010;125:e1178–85. 10.1542/peds.2009-0712 20385628

[5] Landgren V, Svensson L, Gyllencreutz E, et al. Fetal alcohol spectrum disorders from childhood to adulthood: a Swedish population-based naturalistic cohort study of adoptees from eastern Europe. BMJ Open 2019;9:e032407. 10.1136/bmjopen-2019-032407 PMC6830611 31666274

[6] Jarmasz JS, Basalah DA, Chudley AE, et al. Human brain abnormalities associated with prenatal alcohol exposure and fetal alcohol spectrum disorder. J Neuropathol Exp Neurol 2017;76:813–33. 10.1093/jnen/nlx064 28859338 PMC5901082

[7] Gauthier TW, Guidot DM, Kelleman MS, et al. Maternal alcohol use during pregnancy and associated morbidities in very low birth weight newborns. Am J Med Sci 2016;352:368–75. 10.1016/j.amjms.2016.06.019 27776718 PMC5098418

[8] Wozniak JR, Riley EP, Charness ME. Clinical presentation, diagnosis, and management of fetal alcohol spectrum disorder. Lancet Neurol 2019;18:760–70. 10.1016/S1474-4422(19)30150-4 31160204 PMC6995665

[9] Strömland K, Hellström A. Fetal alcohol syndrome--an ophthalmological and socioeducational prospective study. Pediatrics 1996;97:845–50. 8657525

[10] Gummel K, Ygge J. Ophthalmologic findings in Russian children with fetal alcohol syndrome. Eur J Ophthalmol 2013;23:823–30. 10.5301/ejo.5000296 23661538

[11] Andersson Grönlund M, Landgren M, Strömland K, et al. Relationships between ophthalmological and neuropaediatric findings in children adopted from eastern Europe. Acta Ophthalmol 2010;88:227–34. 10.1111/j.1755-3768.2008.01430.x 19416116

[12] Gyllencreutz E, Aring E, Landgren V, et al. Ophthalmologic findings in fetal alcohol spectrum disorders - a cohort study from childhood to adulthood. Am J Ophthalmol 2020;214:14–20. 10.1016/j.ajo.2019.12.016 31926885

[13] Brennan D, Giles S. Ocular involvement in fetal alcohol spectrum disorder: a review. Curr Pharm Des 2014;20:5377–87. 10.2174/1381612820666140205144114 24502600

[14] Ribeiro IM, Vale PJ, Tenedorio PA, et al. Ocular manifestations in fetal alcohol syndrome. Eur J Ophthalmol 2007;17:104–9. 10.1177/112067210701700114 17294389

[15] Moore ES, Ward RE, Wetherill LF, et al. Unique facial features distinguish fetal alcohol syndrome patients and controls in diverse ethnic populations. Alcohol Clin Exp Res 2007;31:1707–13. 10.1111/j.1530-0277.2007.00472.x 17850644

[16] Cook JL, Green CR, Lilley CM, et al. Fetal alcohol spectrum disorder: a guideline for diagnosis across the lifespan. CMAJ 2016;188:191–7. 10.1503/cmaj.141593 26668194 PMC4754181

[17] Watkins RE, Elliott EJ, Wilkins A, et al. Recommendations from a consensus development workshop on the diagnosis of fetal alcohol spectrum disorders in Australia. BMC Pediatr 2013;13:156. 10.1186/1471-2431-13-156 24083778 PMC3849849

[18] Landgraf MN, Nothacker M, Heinen F. Diagnosis of fetal alcohol syndrome (fas): German guideline version 2013. Eur J Paediatr Neurol 2013;17:437–46. 10.1016/j.ejpn.2013.03.008 23618613

[19] Vernescu RM, Adams RJ, Courage ML. Children with fetal alcohol spectrum disorder show an amblyopia-like pattern of vision deficit. Dev Med Child Neurol 2012;54:557–62. 10.1111/j.1469-8749.2012.04254.x 22574626

[20] Popova S, Lange S, Probst C, et al. Estimation of national, regional, and global prevalence of alcohol use during pregnancy and fetal alcohol syndrome: a systematic review and meta-analysis. Lancet Glob Health 2017;5:e290–9. 10.1016/S2214-109X(17)30021-9 28089487

[21] Hemingway SJA, Bledsoe JM, Brooks A, et al. Comparison of the 4-digit code, Canadian 2015, Australian 2016 and Hoyme 2016 fetal alcohol spectrum disorder diagnostic guidelines. Adv Pediatr Res 2019;6:31. 10.35248/2385-4529.19.6.31 31886408 PMC6934106

[22] Coles CD, Gailey AR, Mulle JG, et al. A comparison among 5 methods for the clinical diagnosis of fetal alcohol spectrum disorders. Alcohol Clin Exp Res 2016;40:1000–9. 10.1111/acer.13032 27028727 PMC6346422

[23] Grönlund MA, Aring E, Landgren M, et al. Visual function and ocular features in children and adolescents with attention deficit hyperactivity disorder, with and without treatment with stimulants. Eye 2007;21:494–502. 10.1038/sj.eye.6702240 16518370

[24] Raffa L, Aring E, Dahlgren J, et al. Ophthalmological findings in relation to auxological data in moderate-to-late preterm preschool children. Acta Ophthalmol 2015;93:635–41. 10.1111/aos.12763 26010319

[25] Gill CJ, Sabin L, Schmid CH. Why clinicians are natural bayesians. BMJ 2005;330:1080–3. 10.1136/bmj.330.7499.1080 15879401 PMC557240

[26] Kable JA, O'Connor MJ, Olson HC, et al. Neurobehavioral disorder associated with prenatal alcohol exposure (ND-PAE): proposed DSM-5 diagnosis. Child Psychiatry Hum Dev 2016;47:335–46. 10.1007/s10578-015-0566-7 26202432

[27] Spohr H-L, Willms J, Steinhausen H-C. Fetal alcohol spectrum disorders in young adulthood. J Pediatr 2007;150:179.e1:175–9. 10.1016/j.jpeds.2006.11.044 17236896

[28] Grönlund MA, Andersson S, Aring E, et al. Ophthalmological findings in a sample of Swedish children aged 4-15 years. Acta Ophthalmol Scand 2006;84:169–76. 10.1111/j.1600-0420.2005.00615.x 16637831

[29] Aring E, Grönlund MA, Andersson S, et al. Strabismus and binocular functions in a sample of Swedish children aged 4-15 years. Strabismus 2005;13:55–61. 10.1080/09273970590922664 16020358

[30] Strömland K. Eyeground malformations in the fetal alcohol syndrome. Birth Defects Orig Artic Ser 1982;18:651–5. 6890860

[31] Moutakis K, Stigmar G, Hall-Lindberg J. Using the KM visual acuity chart for more reliable evaluation of amblyopia compared to the hvot method. Acta Ophthalmol Scand 2004;82:547–51. 10.1111/j.1600-0420.2004.00307.x 15453851

[32] Grönlund MA, Aring E, Hellström A, et al. Visual and ocular findings in children adopted from eastern Europe. Br J Ophthalmol 2004;88:1362–7. 10.1136/bjo.2004.042085 15489473 PMC1772390

[33] Andersson Grönlund M, Dahlgren J, Aring E, et al. Ophthalmological findings in children and adolescents with silver-russell syndrome. Br J Ophthalmol 2011;95:637–41. 10.1136/bjo.2010.184457 20805133

[34] Pocock SJ, Simon R. Sequential treatment assignment with balancing for prognostic factors in the controlled clinical trial. Biometrics 1975;31:103–15. 10.2307/2529712 1100130

[35] Twelker JD, Mitchell GL, Messer DH, et al. Children’s ocular components and age, gender, and ethnicity. Optom Vis Sci 2009;86:918–35. 10.1097/OPX.0b013e3181b2f903 19650241 PMC2901932

