# Supplementary material for: The FASD Eye Code: a complementary diagnostic tool in fetal alcohol spectrum disorders
Source: BMJ Open Ophthalmol. 2021 Oct 22;6(1):e000852. doi: 10.1136/bmjophth-2021-000852 (PMC8543669; doi:10.1136/bmjophth-2021-000852)
Supplement: Supplementary data [file bmjophth-2021-000852supp001.pdf]

	**FASD Eye Code Protocol**	
**Name**		
**Code score**	**A. Best corrected visual acuity (BCVA)**	**Patient score**
1	BCVA worst eye logMAR ≤0.1 (decimal ≥0.8)	
2	BCVA best eye logMAR ≤0.5 (decimal ≥0.65)	
3	BCVA each eye/best eye logMAR ≤0.5 and ≥0.3 (decimal ≥0.3 and ≤0.5)	
4	BCVA best eye logMAR >0.5 (decimal <0.3)	
	**B. Refraction in cycloplegia**	
1	No significant refractive error	
2	Hyperopia (≥2.0 D SE) or myopia (≥1.0 D SE) in one or both eyes	
3	Anisometropia (≥1.0 D SE)	
4	Astigmatism (>1.0 D) in one or both eyes	
	**C. Strabismus and binocular function**	
1	Orthophoria or heterophoria and normal stereo acuity (TNO ≤60"; Lang ≤200")	
2	Heterophoria or intermittent heterotropia and normal stereo acuity (TNO ≤60"; Lang ≤200")	
3	No apparent deviation, or heterophoria, or intermittent heterotropia and subnormal stereo acuity (TNO >60"; Lang >200")	
4	Heterotropia and subnormal stereo acuity (TNO >60"; Lang >200") or no stereo acuity (TNO neg; Lang neg)	
	**D. Ocular structural abnormalities**	
1	No ocular structural abnormalities	
2	Ptosis and/or epicanthic folds in one or both eyes	
3	Ocular abnormalities of the retinal vessels (i.e. increased tortuosity) and/or disc (excluding optic nerve hypoplasia)	
4	Optic nerve hypoplasia (ONH in one or both eyes)	
	**Total score (A+B+C+D)**	
