# Supplementary figures and images for: The FASD Eye Code: a complementary diagnostic tool in fetal alcohol spectrum disorders
Source: BMJ Open Ophthalmol. 2021 Oct 22;6(1):e000852. doi: 10.1136/bmjophth-2021-000852 (PMC8543669; doi:10.1136/bmjophth-2021-000852)

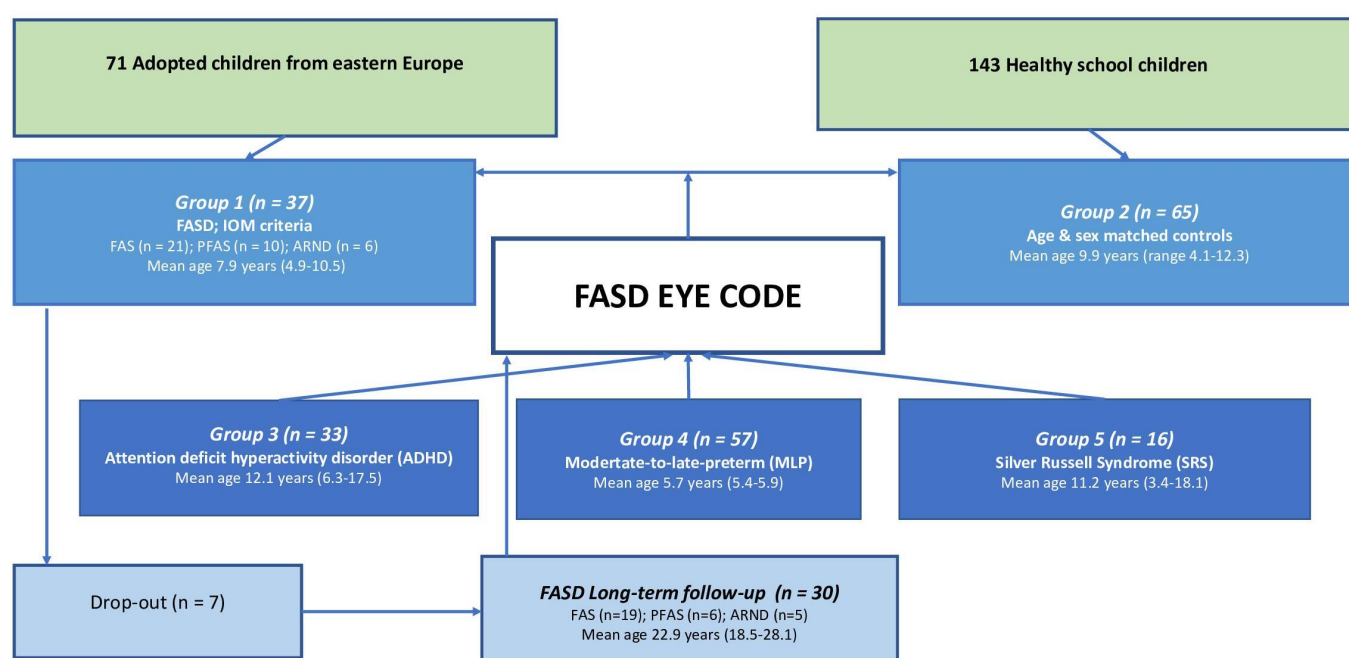

Supplement: Supplementary data [file bmjophth-2021-000852supp002.pdf]
