# Supplementary material for: The FASD Eye Code: a complementary diagnostic tool in fetal alcohol spectrum disorders
Source: BMJ Open Ophthalmol. 2021 Oct 22;6(1):e000852. doi: 10.1136/bmjophth-2021-000852 (PMC8543669; doi:10.1136/bmjophth-2021-000852)
Supplement: Supplementary data [file bmjophth-2021-000852supp003.pdf]

Supplement 3 a–g

3a	FASD vs controls				
Threshold	Specificity	Sensitivity	Accuracy	Positive likelihood ratio	Negative likelihood ratio
-∞	0%	100%	36%	1.0	
4.5	77%	84%	79%	3.6	0.2
5.5	88%	78%	84%	6.4	0.3
6.5	92%	68%	83%	8.8	0.4
7.5	95%	59%	82%	12.9	0.4
8.5	98%	57%	83%	36.9	0.4
9.5	100%	43%	79%	∞	0.6
10.5	100%	27%	74%	∞	0.7
11.5	100%	24%	73%	∞	0.8
12.5	100%	22%	72%	∞	0.8
13.5	100%	16%	70%	∞	0.8
14.5	100%	5%	66%	∞	1.0
∞	100%	0%	64%		1.0

3b	**FAS vs controls**				
**Threshold**	**Specificity**	**Sensitivity**	**Accuracy**	**Positive likelihood ratio**	**Negative likelihood ratio**
-∞	0%	100%	24%	1.0	
4.5	77%	90%	80%	3.9	0.1
5.5	88%	86%	87%	7.0	0.2
6.5	92%	81%	90%	10.5	0.2
7.5	95%	76%	91%	16.5	0.3
8.5	98%	67%	91%	43.3	0.3
9.5	100%	62%	91%	∞	0.4
10.5	100%	43%	86%	∞	0.6
11.5	100%	33%	84%	∞	0.7
12.5	100%	29%	83%	∞	0.7
14	100%	10%	78%	∞	0.9
15.5	100%	5%	77%	∞	1.0
∞	100%	0%	76%		1.0

3c	**FASD vs ADHD**				
Threshold	Specificity	Sensitivity	Accuracy	Positive likelihood ratio	Negative likelihood ratio
-∞	0%	100%	53%	1.0	
4.5	24%	84%	56%	1.1	0.7
5.5	27%	78%	54%	1.1	0.8
6.5	48%	68%	59%	1.3	0.7
7.5	70%	59%	64%	2.0	0.6
8.5	73%	57%	64%	2.1	0.6
9.5	82%	43%	61%	2.4	0.7
10.5	91%	27%	57%	3.0	0.8
11.5	100%	24%	60%	∞	0.8
12.5	100%	22%	59%	∞	0.8
13.5	100%	16%	56%	∞	0.8
14.5	100%	5%	50%	∞	1.0
∞	100%	0%	47%		1.0

3d	**FASD vs MLP**				
Threshold	Specificity	Sensitivity	Accuracy	Positive likelihood ratio	Negative likelihood ratio
-∞	0%	100%	39%	1.0	
4.5	30%	84%	51%	1.2	0.5
5.5	51%	78%	62%	1.6	0.4
6.5	75%	68%	72%	2.8	0.4
7.5	86%	59%	76%	4.2	0.5
8.5	91%	57%	78%	6.5	0.5
9.5	95%	43%	74%	8.2	0.6
10.5	96%	27%	69%	7.7	0.8
11.5	96%	24%	68%	6.9	0.8
12.5	98%	22%	68%	12.3	0.8
13.5	100%	16%	67%	∞	0.8
14.5	100%	5%	63%	∞	1.0
∞	100%	0%	61%		1.0

3e	FASD vs SRS				
Threshold	Specificity	Sensitivity	Accuracy	Positive likelihood ratio	Negative likelihood ratio
-∞	0%	100%	70%	1.0	
4.5	12%	84%	62%	1.0	1.3
5.5	12%	78%	58%	0.9	1.7
6.5	38%	68%	58%	1.1	0.9
7.5	62%	59%	60%	1.6	0.7
8.5	62%	57%	58%	1.5	0.7
9.5	81%	43%	55%	2.3	0.7
10.5	88%	27%	45%	2.2	0.8
11.5	94%	24%	45%	3.9	0.8
12.5	94%	22%	43%	3.5	0.8
13.5	100%	16%	42%	∞	0.8
14.5	100%	5%	34%	∞	1.0
∞	100%	0%	30%		1.0

3f	FASD vs controls, ADHD, MLP and SRS				
Threshold	Specificity	Sensitivity	Accuracy	Positive likelihood ratio	Negative likelihood ratio
-∞	0%	100%	18%	1.0	
4.5	45%	84%	52%	1.5	0.4
5.5	57%	78%	61%	1.8	0.4
6.5	73%	68%	72%	2.5	0.4
7.5	84%	59%	80%	3.7	0.5
8.5	88%	57%	82%	4.8	0.5
9.5	93%	43%	84%	6.1	0.6
10.5	96%	27%	84%	6.8	0.8
11.5	98%	24%	85%	12.0	0.8
12.5	99%	22%	85%	22.0	0.8
13.5	100%	16%	85%	∞	0.8
14.5	100%	5%	83%	∞	1.0
∞	100%	0%	82%		1.0

3g	**FASD vs controls, ADHD and MLP**				
Threshold	Specificity	Sensitivity	Accuracy	Positive likelihood ratio	Negative likelihood ratio
-∞	0%	100%	19%	1.0	
4.5	48%	84%	55%	1.6	0.3
5.5	61%	78%	65%	2.0	0.4
6.5	77%	68%	75%	2.9	0.4
7.5	86%	59%	81%	4.4	0.5
8.5	90%	57%	84%	5.9	0.5
9.5	94%	43%	84%	7.5	0.6
10.5	97%	27%	83%	8.4	0.8
11.5	99%	24%	84%	18.9	0.8
12.5	99%	22%	84%	33.5	0.8
13.5	100%	16%	84%	∞	0.8
14.5	100%	5%	82%	∞	1.0
∞	100%	0%	81%		1.0

∞=infinity

ADHD = Attention Deficit/Hyperactivity Disorders; FAS = Foetal Alcohol Syndrome; FASD = Foetal Alcohol Spectrum Disorders; MLP = Moderate to Late Preterm; SRS = Silver Russell Syndrome.
